# The *in vivo *expression of actin/salt-resistant hyperactive DNase I inhibits the development of anti-ssDNA and anti-histone autoantibodies in a murine model of systemic lupus erythematosus

**DOI:** 10.1186/ar1936

**Published:** 2006-04-10

**Authors:** Anthony P Manderson, Francesco Carlucci, Peter J Lachmann, Robert A Lazarus, Richard J Festenstein, H Terence Cook, Mark J Walport, Marina Botto

**Affiliations:** 1 Rheumatology Section, Division of Medicine, Faculty of Medicine, Imperial College, London, UK; 2 Institute of Molecular Biosciences, The University of Queensland, Brisbane, 4072, Australia; 3 Department of Veterinary Medicine, University of Cambridge, Cambridge, UK; 4 Department of Protein Engineering, Genentech, Inc., CA, USA; 5 Gene Control Mechanisms and Disease, Imperial College, London, UK; 6 Department of Histopathology, Faculty of Medicine, Imperial College, London, UK; 7 The Wellcome Trust, London, UK

## Abstract

Systemic lupus erythematosus (SLE) is characterised by the production of autoantibodies against ubiquitous antigens, especially nuclear components. Evidence makes it clear that the development of these autoantibodies is an antigen-driven process and that immune complexes involving DNA-containing antigens play a key role in the disease process. In rodents, DNase I is the major endonuclease present in saliva, urine and plasma, where it catalyses the hydrolysis of DNA, and impaired DNase function has been implicated in the pathogenesis of SLE. In this study we have evaluated the effects of transgenic over-expression of murine DNase I endonucleases *in vivo *in a mouse model of lupus. We generated transgenic mice having T-cells that express either wild-type DNase I (wt.DNase I) or a mutant DNase I (ash.DNase I), engineered for three new properties – resistance to inhibition by G-actin, resistance to inhibition by physiological saline and hyperactivity compared to wild type. By crossing these transgenic mice with a murine strain that develops SLE we found that, compared to control non-transgenic littermates or wt.DNase I transgenic mice, the ash.DNase I mutant provided significant protection from the development of anti-single-stranded DNA and anti-histone antibodies, but not of renal disease. In summary, this is the first study *in vivo *to directly test the effects of long-term increased expression of DNase I on the development of SLE. Our results are in line with previous reports on the possible clinical benefits of recombinant DNase I treatment in SLE, and extend them further to the use of engineered DNase I variants with increased activity and resistance to physiological inhibitors.

## Introduction

Systemic lupus erythematosus (SLE) is a disease characterized by the production of a variety of auto-antibodies against ubiquitous intracellular and cell surface antigens. Detailed analysis of these autoantibodies by many researchers has revealed several key findings. First, nuclear antigens are prominent with anti-double-stranded DNA (dsDNA) and anti-nucleosome antibodies extremely common in SLE patients (reviewed in [1]). Second, immune complexes containing these autoantibodies and nucleosomes are thought to mediate pathology following their localization in tissues [2-4]. Third, the anti-nuclear antibodies demonstrate all the hallmarks of an antigen-driven, T-cell dependent mechanism [5]. The antibodies are of high affinity, have undergone isotype switching and show evidence of somatic mutation and epitope spreading [6].

Accumulating evidence suggests that inefficient clearance of apoptotic cells provide the source of the nuclear antigens driving the development of autoimmunity. The autoantigens targeted in SLE have been shown to cluster in and on the surface blebs of apoptotic cells [7,8] and ablation in mice of a number of genes whose products mediate the clearance of apoptotic cells, such as C1q [9,10], secreted IgM [11,12], cMer [13,14] and transglutaminase 2 [15,16] is associated with the development of a lupus-like disease.

DNase I catalyses the hydrolysis of dsDNA, whether free or as part of a nucleosome, and is the major endonuclease present in saliva, urine and plasma in mice [17,18]. Impaired DNase I function has been implicated in the pathogenesis of SLE for many years since the initial observation that DNase activity is low in the serum of patients with SLE [19] and in lupus-prone NZB/NZW mice [20]. The reduced DNase I activity in SLE patients also correlates with an increased serum concentration of globular actin (G-actin), a potent inhibitor of DNase [19,21]. Mutations in *Dnase1 *have been identified in two Japanese SLE patients, resulting in low DNase activity and severe disease [22]. However, two subsequent studies failed to identify *Dnase1 *mutations among SLE patients of different ethnic origin [23,24]. Of note, mice lacking DNase I (*Dnase1*-/-) have been shown to develop a spontaneous lupus-like syndrome [25]. These observations led to the speculation that DNase I may regulate disease progression by degrading DNA released from dying cells, thereby reducing the antigen load driving the immune response, and by facilitating the hydrolysis of circulating and/or deposited DNA-antibody complexes [26].

There is already evidence that exogenous administration of DNase may have some therapeutic activity in mice and humans. On treating patients with SLE with bovine DNase I, clinical responses were observed in six patients and three of them showed a reduction in their levels of anti-DNA antibodies [27] More recently, a phase 1b study of the use of recombinant human DNase I in patients with SLE demonstrated that the treatment was safe. No change in serum markers of disease were observed, however, perhaps due to the fact that catalytically active levels of the enzyme in the circulation were achieved only for very brief periods [28]. In mice, the data have been mixed, with Macanovic and colleagues [29] finding that subcutaneous injection of recombinant DNase I led to significant disease improvement in NZB/NZW mice, especially if the DNase was administered during the most active stage of disease. However, in a second study, Verthelyi and colleagues [30] reported that intraperitoneal injection of DNase I in young NZB/NZW mice did not delay the onset, or reduce the severity, of glomerulonephritis, or prolong survival. One of the problems faced by both of these studies is that G-actin, a potent inhibitor of DNase I activity, is present at high levels in both SLE patients and NZB/NZW mice [19,21]. To address this issue Lazarus and colleagues [31,32] have generated and characterized a number of DNase I mutants, including ones resistant to inhibition by G-actin. Mutations were also introduced to increase the affinity of the DNase I for DNA, resulting in two improved characteristics – increased specific activity compared to the wild-type enzyme and elimination of the inhibition by salt at physiological concentrations [31-34]. To test the hypothesis that DNase I *in vivo *might protect from the development of anti-nuclear antibodies and associated pathology by reducing the circulating levels of antigenic nuclear components, we have taken advantage of the mutant murine DNase I constructs to generate transgenic mice over-expressing either wild-type (wt.DNase I) or the actin/salt-resistant hyperactive mutant (ash.DNase I) protein. DNase transgenic mice were inter-crossed with mice lacking serum amyloid P component (*Apcs*-/-), previously shown to develop high titres of anti-nuclear antibodies and glomerulonephritis [35,36], and the phenotypes of the different transgenic mice in the presence or absence of serum amyloid P component were compared. In this model, a mild protective effect of DNase I was observed, with lower anti-single-stranded DNA (ssDNA) and anti-histone antibody levels in transgenic mice compared to littermate controls. In addition, in mice treated with lipopolysaccharide (LPS), which induces a transient pulse of plasma nucleosomes and DNA [37,38], a significant reduction in the level of circulating DNA was observed in ash.DNase I transgenic mice. These data demonstrate that the therapeutic use of a recombinant actin-resistant, salt-resistant and hyperactive DNase I has potential to alter the development of autoimmunity.

## Materials and methods

### Construction of DNase transgenic mice

The cDNA encoding murine wt.DNase I and a murine hyperactive mutant resistant to inhibition by both salt and actin (ash.DNase I) were made using methods previously described [30-33]. The cDNA for the murine ash.DNase I contained codons CGG:AAA at residues 13:205 and CGT at residue 114. This resulted in mutations E13R:T205K, which enhance activity and impair inhibition by salt, and A114R to eliminate inhibition by G-actin [30-33]. The cDNAs were inserted into the human pVA vector (gift from Professor D Kioussis, National Institute for Medical Research, London), which contains the human CD2 control region [39,40]. The constructs were excised from the vectors by digestion with Kpn I and Not I, and purified using a QIAEX II gel extraction kit (Qiagen, Crawley, UK) followed by Elutip purification (Schleicher and Schuell, London, UK). The DNA was injected into fertilized CBA × C57BL/6 F1 mouse eggs and these were transplanted into foster females. Progeny were screened for transgene integration by slot blotting, using the human CD2 sequence as a probe, and by PCR. Expression of transgenic DNase I was measured in two ways: secretion of DNase from T cells purified from transgenic mice, as described below; and measuring DNase activity in urine and serum by DNA-methyl green (DNA-MG) assay, as described below. Several transgenic lines were generated and the ones with the highest DNase I activity were selected for further breeding. Both transgenic lines (wt.DNase I and ash.DNase I) were backcrossed to the C57BL/6 genetic background for six generations before use.

### Experimental cohorts

For studying spontaneous autoimmunity, the transgenic mice were inter-crossed with *Apcs*-/-mice [35,36,41]. Cohorts of more than 20 female mice per group (C57BL/6 (*n *= 45), *Apcs*-/- (*n *= 44), wt.DNase I (*n *= 30), ash.DNase I (*n *= 25), wt.DNase I.*Apcs*-/- (*n *= 21), ash.DNase I.*Apcs*-/- (*n *= 21)) were generated. Importantly, the control mice (C57BL/6 and *Apcs*-/-) were littermates of the transgenic animals. Serum samples were collected from all mice monthly from 3 months of age, until sacrifice at 12 months, and stored at -80°C until use. Animals were kept under specific pathogen-free conditions. All animal care and procedures were conducted according to institutional guidelines.

### Serological analyses

Levels of IgG anti-nuclear antibodies were assessed by indirect immunofluorescence using Hep-2 cells and a fluorescein-conjugated IgG Fc-specific anti-mouse Ab (Sigma-Aldrich, Dorset, UK). Serum samples were screened at a 1:80 dilution in PBS supplemented with 2% BSA, 0.05% Tween 20, 0.02% NaN_3 _and the positive samples titrated to end point.

Total IgG antibodies to ssDNA and anti-chromatin antibodies were measured by ELISA as described previously [42]. Briefly, plates were coated with ssDNA (10 μg/ml) or chromatin (0.5 mg/ml) for 3 hours at 37°C. Serum samples were screened at 1:100 and 1:300 dilution for anti-ssDNA and anti-chromatin antibodies, respectively. Bound antibodies were detected with AP-conjugated goat anti-mouse IgG (γ-chain specific; Sigma-Aldrich), and the results were expressed in arbitrary ELISA units (AEU) relative to a standard positive sample derived from an MRL/Mp.*lpr/lpr *mice pool.

Total IgG antibodies to dsDNA were measured by ELISA as previously described [43]. Briefly, plates were coated with 1 μg/mL streptavidin (Sigma-Aldrich), incubated overnight at 4°C and post-coated with PBS supplemented with 0.5% BSA. φX174 double-stranded plasmid DNA (Promega, Southhampton, UK) was biotinylated with Photoprobe biotin (Vector Laboratories, Petersborough, UK), then added to the streptavidin plate at 200 ng/ml and incubated overnight at 4°C. Serum samples were assayed at 1:100 dilution, in triplicate with one well per sample containing no dsDNA to allow determination of the non-specific binding to streptavidin. Bound antibodies were detected and quantified as above.

Total IgG antibodies to histone were also measured by ELISA. Plates were coated with calf thymus histone (5 μg/ml; Calbiochem, Nottingham, UK) overnight at 4°C. Serum samples were screened at 1:100 dilution; bound antibodies were detected and quantified as above.

Total IgM antibodies to ssDNA were measured by ELISA as for IgG above, except serum samples were diluted 1:500 and bound antibodies were detected with AP-conjugated goat anti-mouse IgM (Sigma-Aldrich).

### Renal histology and immunohistochemistry

Kidneys were fixed in Bouin's solution for at least 2 hours, transferred into 70% ethanol, and processed into paraffin. The sections were stained with periodic acid-Schiff reagent and scored for glomerulonephritis. Glomerular hypercellularity was ranked in a blinded fashion as follows: grade 0, normal; grade I, hypercellularity involving greater than 50% of the glomerular tuft in 25% to 50% of glomeruli; grade II, hypercellularity involving greater than 50% of the glomerular tuft in 50% to 75% of glomeruli; grade III, hypercellularity involving greater than 75% of the glomeruli or crescents in greater than 25% of glomeruli; grade IV, severe proliferative glomerulonephritis in greater than the 90% of glomeruli.

For quantitative immunofluorescence, fluorescein isothiocyanate (FITC)-conjugated goat antibodies against mouse total IgM, IgG (Sigma-Aldrich) and C3 (ICN Pharmaceuticals, Basingstoke, UK) were used on snap-frozen sections. The staining with FITC-conjugated antibodies was quantified as previously described [44] and expressed as arbitrary fluorescence units. The analysis was done blind and 50 glomeruli per section were analysed.

### Flow cytometry

Flow cytometry was performed using a three colour staining on spleen cells and thymocytes and analysed with a FACSCalibur™ (Becton-Dickinson, Mountain View, CA, USA). The following antibodies were used: anti-CD90.2 (53-2.1), anti-B220 (RA3-6B2), anti-CD5 (53-7.3), anti-CD19 (1D3), anti-CD25 (PC61), anti-CD69 (H1.2F3), anti-CD62L (MEL-14), anti-CD44 (IM7), anti-CD4 (RM4-5) and anti-CD8 (53-6.7). All antibodies were purchased from Pharmingen-Becton Dickinson (San Diego, CA, USA). Biotinylated antibodies were detected using an allophycocyanin-conjugated streptavidin antibody (Pharmingen-Becton Dickinson). Staining was performed in the presence of saturating concentration of 24G2 monoclonal antibody (anti-FcγRII/III). Data were analysed using WinMDI software (version 2.8, Scripps Institute, USA).

### *In vitro *T-cell stimulation

Ninety-six-well microtitre plates (Nunc, Rochester, NY, USA) were pre-coated overnight at 4°C with anti-CD3 (Pharmingen-Becton Dickinson) diluted in PBS to 10, 3.3 and 1 μg/ml. Before use, the plates were washed three times with PBS to remove unbound antibody. Lymph nodes were removed from transgenic mice and single cell suspensions prepared under sterile conditions in RPMI containing 10% heat-inactivated fetal calf serum, 100 μg/ml streptomycin, 100 units/ml penicillin, 2 mM glutamine and 50 μM 2-mercaptoethanol. Finally, cells were resuspended to 10^6^, 3 × 10^5^, and 10^5 ^cells/ml and 200 μl of each added to appropriate wells in uncoated or anti-CD3 coated plates and incubated for 72 hours at 37°C. DNase activity in the supernatants was measured by DNA-MG assay, as set out below.

### DNA-methyl green assay

DNase I activity in urine and supernatant samples was quantified using the colorimetric DNA-MG assay previously described [45-47]. Mice were placed in metabolic cages for 24 hours to allow collection of urine. Calf-thymus DNA (Sigma-Aldrich) was dissolved to 2 mg/ml in HEPES/EDTA buffer (25 mM HEPES, 1 mM EDTA, pH 7.5) and methyl green dye (Sigma-Aldrich) was dissolved to 0.4% in 20 mM Na-acetate buffer, pH 4.2. To prepare the DNA-MG substrate, the dissolved dye (0.4%) was mixed with the calf-thymus DNA (2 mg/ml) to make a final dye concentration of 0.02%. Multiple dilutions of samples were made in MG-assay buffer (25 mM HEPES, 4 mM CaCl_2_, 4 mM MgCl_2_, 0.1% BSA, 0.05% Tween 20, 0.05% NaN_3_, pH 7.5), mixed 1:1 with DNA-MG in a 96-well microtitre plate (Nunc) and then incubated for 15 hours at 37°C. The absorbance was read in a plate reader using a 620 nm filter. DNase activity in the samples was calculated based on a standard curve of recombinant bovine DNase I (Sigma-Aldrich). In addition to the standard assay buffer, optimised to generate maximal DNase activity, inhibitors of wild-type DNase were added to the buffer. To measure the level of activity in the presence of physiological salt, 0.9% NaCl was added to the assay buffer and DNA-MG reagent. The DNA-MG reagent was left overnight before use to allow for re-equilibration of the dye. Samples were diluted in the buffer containing NaCl for at least 30 minutes prior to addition of the DNA substrate. To measure inhibition of DNase I activity by G-actin, 0.4 mM ATP (Sigma-Aldrich) was added the sample buffer, in addition to various concentrations of G-actin (1 to 1000 μg/mL; Sigma-Aldrich). Samples were diluted in the buffer containing G-actin/ATP for at least 30 minutes prior to addition of the DNA substrate.

### Lipopolysaccharide model

Female C57BL/6, wt.DNase I and ash.DNase I were injected intraperitoneally with a single dose of 100 μg LPS from *Escherichia coli *0111:B4 (Sigma-Aldrich). Mice were bled from the tail vein before, and 9 hours, 7 days and 14 days post-injection of LPS.

Blood samples were collected into EDTA (final concentration 20 mM), centrifuged to separate red blood cells and the plasma stored at -80°C until use. The level of circulating DNA was assessed by a PicoGreen (Molecular Probes, Eugene, OR, USA) fluorimetric assay, as per the manufacturer's instructions and as previously described [37,48]. Briefly, serial plasma samples were made in TE buffer (10 mM Tris, 1 mM EDTA, pH 8) and mixed at a 1:1 ratio with the PicoGreen reagent, diluted 1:200 in TE buffer, in a 96-well microtitre plate (Nunc). Fluorescence was measured using a Wallac Victor fluorescence plate reader (Perkin-Elmer, Groningen, The Netherlands) with an excitation wavelength at 485 nm and an emission wavelength at 520 nm.

### Statistics

The data are presented as mean ± standard error of the mean (SEM), unless otherwise stated. The non-parametric Mann-Whitney test was used to compare two groups and the Kruskal-Wallis test with Dunn's multiple comparison test for analysis of three groups, with differences being considered significant for *p *values < 0.05. Statistics were calculated using GraphPad Prism version 3.0 (GraphPad Software, San Diego, CA, USA).

## Results

### Generation of DNase I transgenic lines

The cDNAs encoding murine wt.DNase I or the E13R:A114R:T205K mutant DNase I (ash.DNase I), engineered for resistance to inhibition by G-actin and physiological salt as well as increased hydrolytic activity, were inserted into the human CD2 Locus Control Region vector [39] that has previously been shown to be effective in overcoming chromosomal position-dependent gene repression of heterologous genes, allowing the production of the transgene in a predictable copy-number dependent manner [40]. The CD2 control region directs expression of the transgene exclusively to the T cell compartment. Mice containing the *Dnase1 *transgenes were determined by Southern blot analysis, probing for the human CD2 promoter elements encoded by the vector (data not shown). For each construct, several lines of transgenic mice were initially generated and those containing the highest copy number were selected for subsequent backcrossing onto the C57BL/6 strain for 6 generations. Semi-quantitative slot blotting analysis showed that approximately 10 copies of the transgene were present in wt.DNase I, whilst the ash.DNase I mice contained eight copies (data not shown). The DNase sequences were PCR amplified from the transgenic mice, and the products subjected to specific restriction digests and sequenced (data not shown) to confirm the presence of the wt.DNase I or ash.DNase I construct.

**Figure 1:**
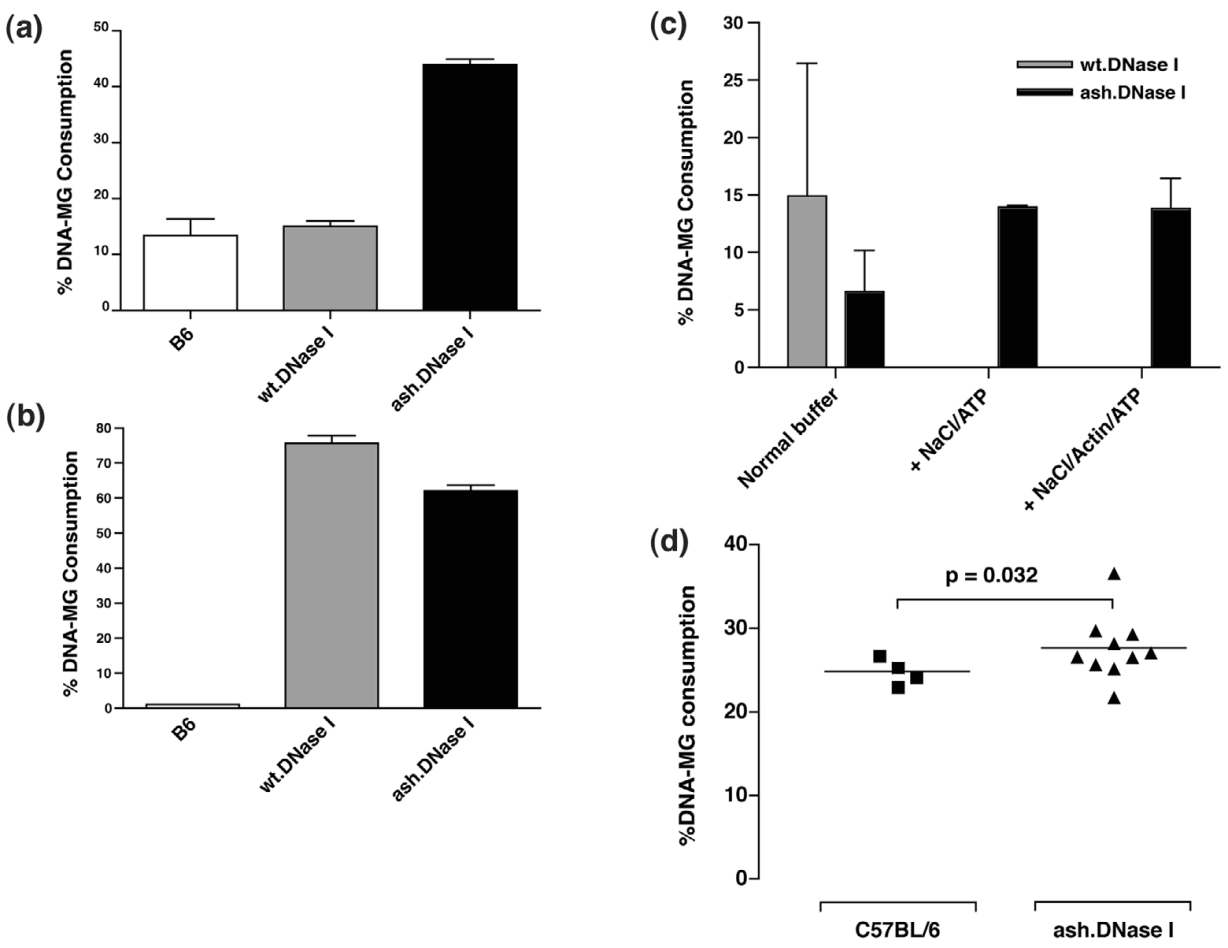
DNase I production in transgenic mice. **(a) **DNase secretion from lymph node cells was quantified by DNA-methyl green (DNA-MG) assay. Cells were placed in culture for 3 days either in (a) the absence or **(b) **presence of anti-CD3 antibodies. Supernatants were diluted in assay buffer 1:2 for unstimulated cells and 1:16 for stimulated cells. **(c) **Activity of the DNase I present in the supernatants purified as in (b). The activity was determined in the presence of the normal DNA-MG assay buffer, described in Materials and methods, or following the addition of 0.9% NaCl/ATP or NaCl/ATP/Actin. The level of DNase I activity in the supernatants was adjusted to give similar levels in the normal DNA-MG assay buffer. **(d) **DNase I activity in the urine of actin-resistant, salt-resistant and hyperactive mutant of DNase I (ash.DNase I) transgenic mice compared to control C57BL/6 littermates. wt.DNase I, wild-type DNase I.

To confirm the expression and secretion of DNase I from the T cells in the transgenic mice, the lymph nodes were removed, and single cell suspensions prepared and placed in culture *in vitro*. In the absence of stimuli, the transgenic T cells only secrete low levels of DNase I (Figure 1a); however, following activation of the T cells by the pre-coating of the plate with anti-CD3 antibodies (Figure 1b) or Concanavalin A (data not shown), a large increase in the level of DNase I secreted into the supernatant was observed compared to the level in the supernatant from T cells of non-transgenic littermate mice. To confirm the functional activity of the secreted DNase I, the assay was also carried out in the presence of physiological salt concentrations and G-actin. As expected, the DNase I secreted from T cells purified from the wt.DNase I transgenic mice was inhibited in the presence of 0.9% NaCl and G-actin, but the mutant DNase I secreted from T cells isolated from ash.DNase I mice was resistant to inhibition by NaCl and G-actin (Figure 1c).

A significant increase in total DNase activity was also observed in the 24 hour urine collection from transgenic mice compared to their non-transgenic littermates (Figure 1d). DNase activity was mainly measured from urine instead of serum because the absence in urine of natural inhibitors of DNase activity such as G-actin.

To test if the expression of DNase I from T cells in the transgenic mice had caused alterations in T-cell development or introduced aberrations in the mature peripheral populations, a comprehensive flow cytometry analysis was performed. Three-colour analysis of the splenic and thymic cell populations did not highlight any significant abnormalities (Table 1). Most importantly, between the transgenic mice and littermate controls, the percentage of CD4+CD8- cells within the thymus (3.9 ± 0.4 and 3.6 ± 0.3, respectively), and the percentage of naïve T cells within the periphery (72.8 ± 3.1 and 73.2 ± 2.6, respectively) were identical.

### LPS model of autoimmunity

**Figure 2:**
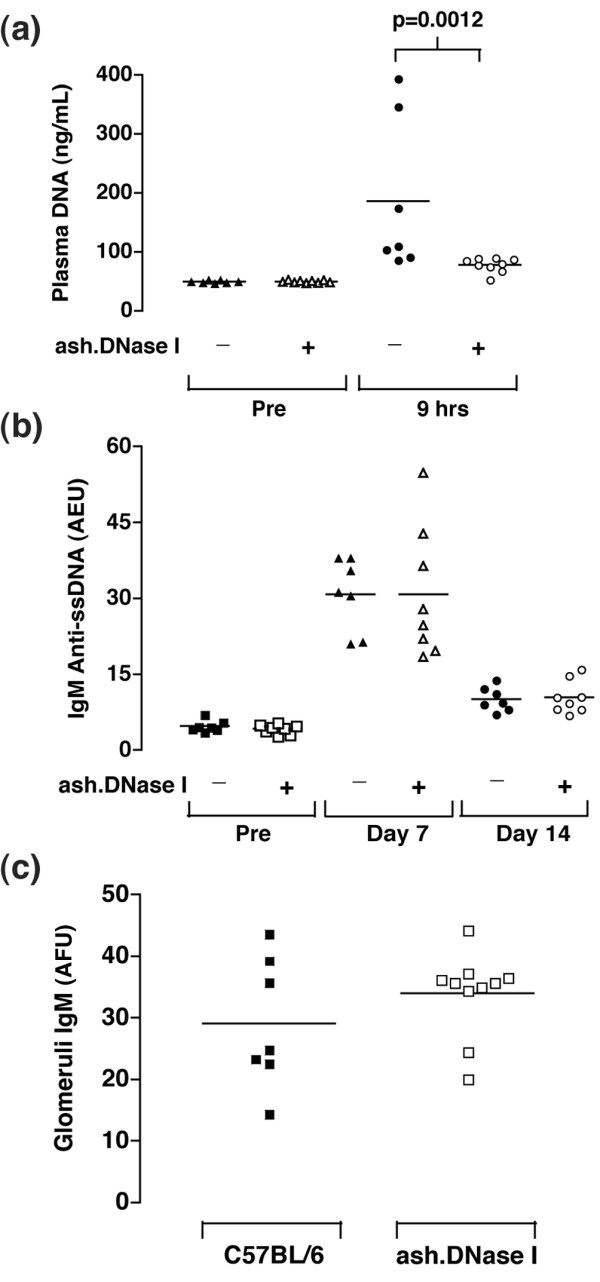
Transient autoimmunity was induced in female C57BL/6 and actin-resistant, salt-resistant and hyperactive mutant of DNase I (ash.DNase I) mice by intraperitoneal injection with a single dose of 100 μg lipopolysaccharide (LPS; *E. coli *0111:B4). Serum and plasma samples were collected from mice before, and 9 hours, 7 days and 14 days post-LPS injection. **(a) **Plasma DNA concentration was determined by fluorometric assay with the dye PicoGreen, as described in the Materials and methods. Assays were performed in triplicate and values represent the mean results from each mouse. **(b) **Levels of serum IgM anti-ssDNA antibodies in mice before and 7 days and 14 days post-injection of LPS. Values are expressed in arbitrary ELISA units (AEU) related to a standard positive sample that was assigned a value of 100 AEU. **(c) **The level of IgM deposited in the glomeruli was quantified by immunofluorescence and expressed as arbitrary fluorescence units (AFU). The data are representative of four separate experiments.

Transient autoimmunity can be induced in mice by the administration of a single high dose of LPS. This induces a pulse of nucleosomes and chromatin in the plasma within the first 12 hours post-injection accompanied by an increase in circulating anti-DNA antibody levels and immune-complexes deposited in the kidneys [37,38,49,50]. To investigate if the increased expression of DNase I could provide any protective effect in this model, a single dose of 100 μg LPS (*E. coli *0111:B4) was injected intraperitoneally into DNase transgenic mice and their non-transgenic littermates. This protocol was established following testing of a number of different forms of LPS, and a wide range of doses, for their ability to induce a strong immunological response without causing excessive toxicity to the mice. Figure 2a shows that at 9 hours post-injection of LPS a large increase in circulating DNA can be measured in the plasma of non-transgenic mice, although significantly lower levels of circulating DNA were observed in plasma from ash.DNase I transgenic mice. This observation clearly demonstrates that the level of DNase activity is functionally higher in the ash.DNase I transgenic mice. No protective effect was observed in the wt.DNase I transgenic mice compared to their littermate controls (data not shown), most likely because the increased activity was neutralised by the raised level of G-actin.

Circulating IgM anti-ssDNA antibodies were present in all mice by day 7 post-injection of LPS and were still present at day 14. No significant differences were observed in the either the wt.DNase I or ash.DNase I transgenic mice compared to non-transgenic mice (Figure 2b). No IgG anti-ssDNA antibodies were detected in any mice. The kidneys were isolated from all mice 14 days after the administration of LPS, and sections stained for total IgM, IgG and C3. A significant increase in the level of IgM deposited within the glomeruli was observed in all mice when compared to non-infected mice. However, no significant protective effect was observed in the ash.DNase I transgenic mice (Figure 2c). The levels of glomerular C3 and IgG deposition did not change following the LPS-injection.

### Spontaneous autoimmunity

**Figure 3:**
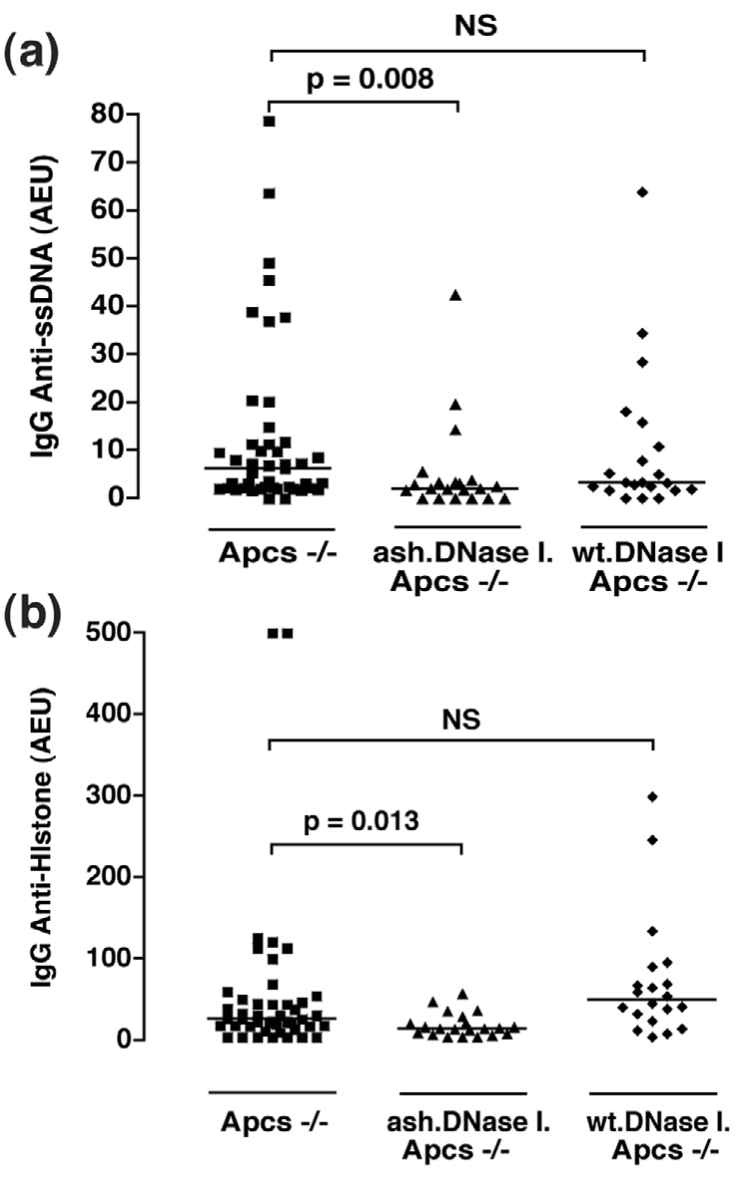
Autoantibody profiles of the experimental cohorts at 12 months of age. **(a) **IgG anti-ssDNA titres in the serum of *Apcs*-/-, wt.DNase I. *Apcs*-/- and ash.DNase I. *Apcs*-/- mice. Values are expressed in arbitrary ELISA units (AEU) related to a standard positive sample that was assigned a value of 100 AEU. Each symbol represents one mouse. **(b) **Anti-histone titres in the same groups of mice as in (a), and statistics were performed as described in the Materials and methods with significant differences if *p *< 0.05. Error bars indicate standard error of the mean. NS, not significant.

We have previously reported that *Apcs*-/- mice on the C57BL/6 genetic background spontaneously develop a lupus-like disease [35,36]. Therefore, to study the role of DNase I in the development of spontaneous autoimmunity, we inter-crossed the DNase transgenic mice on the C57BL/6 genetic background with *Apcs*-/- (backcrossed onto C57BL/6 for 10 generations) mice and generated the following cohorts: wt.DNase I, ash.DNase I, wt.DNase I.*Apcs*-/-, ash.DNase I.*Apcs*-/-, C57BL/6 and *Apcs*-/-. Blood samples were collected from mice at different time points and screened for the presence of a variety of autoantibodies. Figure 3 and Table 2 show the final results at 12 months of age when all the animals were sacrificed. IgG anti-ssDNA (Figure 3a) and anti-histone (Figure 3b) antibodies were significantly lower in the ash.DNase I.*Apcs*-/- mice compared to both *Apcs*-/- and wt.DNase I.*Apcs*-/- animals. This protective effect of ash.DNase I on the generation of anti-ssDNA autoantibodies was already present at six months of age (ash.DNase I.*Apcs*-/- = 11.0 ± 4.2 AEU; *Apcs*-/- = 23.5 ± 7.2 AEU; *p *= 0.03). A trend towards lower IgG anti-dsDNA antibody levels was also observed in the ash.DNase I.*Apcs*-/- mice compared to control animals, although this did not reach significance (Table 2). Anti-chromatin and anti-nuclear antibody titres were the same in all three cohorts of mice (Table 2). It is noteworthy that no serological differences were observed between the wt.DNase I.*Apcs*-/- and the *Apcs*-/- mice at any of the time points analysed. As expected, only very low levels of autoantibodies were detected in any of the remaining cohorts (wt.DNase I, ash.DNase I and C57BL/6; data not shown). The histological assessment of the kidneys failed to reveal any significant beneficial effect of the DNase transgenes on the development of lupus-nephritis (Table 3).

## Discussion

There is strong evidence for a role of DNase I in SLE with DNase activity low in SLE patients and SLE-prone mice, and *Dnase1*-/- mice developing a spontaneous lupus-like phenotype. Here we have shown that the transgenic expression of mutant DNase I (ash.DNase I), conferring resistance to the natural inhibitors of DNase I and having increased activity, can provide some protective effect against the development of autoantibodies in mice. However, the reduction in the autoantibody level obtained in our experimental model was not sufficient to prevent the mice from developing lupus nephritis.

Several different methods have been developed to measure DNase I activity [51,52]. Measurement is difficult under physiological conditions due to its low concentration in plasma, low activity at physiological salt, pH dependence, and inhibition by G-actin [19,20,46,47,51]. In our hands, the DNA-MG assay proved to be consistent and reliable for screening the DNase levels in mice. In healthy transgenic mice, modest increases in the level of total DNase activity were obtained compared to their littermate controls, even with high copy numbers of the transgenes. The T cell stimulation assays demonstrate that following activation the transgenic T cells were able to secrete large amounts of bioactive DNase; however, in the absence of activation, the transgenic T cells only secreted low levels. Therefore, the modest increases detected in biological fluids (plasma and urine) probably reflected a general state of inactivation of the peripheral T cells in healthy mice.

Previous studies have shown that DNA in the blood most likely circulates in the form of nucleosomes that are released from dead or dying cells [38], and is not freely soluble but in complex with other blood proteins [53-55]. The poor clearance and subsequent immunogenicity of these DNA complexes is thought to be central to the pathogenesis of SLE. Thus, we felt that it was important initially to demonstrate increased consumption of these complexes *in vivo *in our transgenic mice. Following the challenge of the transgenic mice with a high dose of LPS, a significant reduction in the circulating levels of potentially immunogenic DNA was obtained in the ash.DNase I mice. This result clearly demonstrates that functionally the transgenic DNase was active *in vivo *despite only modest increases in total DNase activity detected in these mice. The PicoGreen assay used to detect the circulating DNA is extremely sensitive and has been shown to allow quantification of soluble DNA as well as that bound within immune complexes, and is not affected by the length of the DNA [48,56]. It requires dsDNA to intercalate, however, and, therefore, any circulating ssDNA could not be detected.

A number of groups have previously reported that the challenge of mice with a high dose of LPS leads to a transient autoimmunity characterised by the development of a range of autoantibodies of both the IgM and IgG isotypes, and subsequent glomerular disease [37,38,49,50]. In our hands, high levels of IgM anti-ssDNA antibodies were induced in all mice following the injection of LPS, although no subsequent development of IgG anti-ssDNA antibodies was observed. In contrast to the profound protective effect of ash.DNase I expression on the level of circulating DNA, no protection against the development of the IgM anti-dsDNA antibodies was provided. In view of the lack of IgG anti-ssDNA antibodies observed in our experimental model, we believe that the increase in IgM anti-ssDNA antibodies reflected a general induction of B cell activation by LPS, and not an antigen driven development of autoantibodies. In addition, we did not observe any pathology in the kidneys of the mice, with the exception of a general increase in deposited IgM. The sensitivity and response to LPS has been shown to vary greatly depending on the mouse strain and the type of LPS [49] used. Therefore, the lower immunological response observed in our hands may reflect differences in the experimental conditions applied.

Mice defective in a number of genes have provided important insights into human SLE, presenting with similar disease phenotypes and leading to the identification of orthologous genes or similar families of genes that drive disease in humans. We and others have reported that deficiency in serum amyloid P component leads to development of a spontaneous lupus-like phenotype in mice [35,36]. Serum amyloid P component is known to bind to chromatin [57-59] and apoptotic cells [60-62]. Therefore, in its absence, it has been proposed that the impaired removal of chromatin from the circulation is responsible for the development of the lupus-like disease observed in *Apcs*-/- mice [35,36]. These observations made *Apcs*-/- mice an ideal model to test whether increasing the concentration and function of DNase I can protect mice from developing autoimmunity. However, recently it has emerged that not all of the autoimmune features observed in these mice is mediated by deficiency of the *Apcs *gene [63]. Additional polygenic genes in the region surrounding the knockout construct, a locus on chromosome 1 known to be a hotspot for genes implicated in SLE, also contribute to the development of disease. Despite these confounding factors, *Apcs*-/- mice on the C57BL/6 genetic background spontaneously develop a lupus-like disease and, therefore, still represent a suitable model to test the role of DNase I in the disease process. Importantly, to minimise any epistatic effect, non-transgenic littermates were used as controls in this study.

In SLE, a huge range of autoantigens are targeted, although it is complexes containing DNA and nucleosomes that are thought central to pathogenesis. Tracking the development of a range of autoantibodies from three months of age in the cohorts revealed protection from the development of anti-ssDNA and anti-histone in the ash.DNase I transgenic mice, but only a non-significant decrease in the level of anti-dsDNA, anti-nuclear or anti-chromatin antibodies. A possible explanation for this is that, in these complexes, the DNA is present in a different form, less susceptible to DNase digestion [48]. In this context, it is of note that Macanovic and colleagues [29] treated NZB/NZW mice with recombinant DNase I and observed a reduction in the titre of anti-DNA but not anti-cardiolipin antibodies. In addition, a recent report has demonstrated that plasminogen co-operates with human and mouse DNase I in the breakdown of chromatin within necrotic cells [64]. The importance of this interaction in the overall function of DNase I, especially with respect to removal of potentially immunogenic chromatin complexes *in vivo*, remain to be confirmed, and it is possible that other related serine proteases may also mediate this process. Thus, it is possible that the level of additional accessory proteins present in our mice may have limited the effectiveness of DNase I *in vivo*. Furthermore, the significance of anti-DNA antibodies binding to DNase leading to inhibition of its activity is still unclear [27,29,65] and was not directly tested in this study. Thus it is possible that some anti-dsDNA antibodies might have limited the DNase I activity *in vivo*. Finally, the reason only mild protection from the development of autoantibodies was observed in the ash.DNase I transgenic mice may simply reflect the modest expression levels obtained in these mice.

Mild glomerulonephritis was observed in all serum amyloid P component-deficient cohorts, with no protection provided by the over-expression of DNase I. As significantly lower levels of anti-ssDNA antibodies were observed in the ash.DNase I transgenic mice, one could conclude that in our experimental model these autoantibodies were not critical in the early stages of the renal disease. These findings are consistent with data reported by Verthelyi and colleagues [30], who observed lowered numbers of B cells secreting anti-DNA antibodies in NZB/NZW mice treated with recombinant DNase I, but no alteration to the renal function or overall clinical outcome. In another study, however, significant improvement in the renal pathology was reported, especially if the recombinant DNase I was administered during the most active stage of disease [29]. The discrepancy between the two studies could be due to a number of factors, including the longevity of the injected protein and the level of free G-actin in the circulation at sites of inflammation inhibiting the DNase I activity. Our data obtained by a transgenic expression of the mutant protein (ash.DNase I) in T cells appear to favour the hypothesis that DNase I cannot alter the initial stages of glomerular disease. However, as only mild renal disease developed in the mice used in our study, we were unable to shed any light on the role of DNase I in the more advanced stages of lupus nephritis. It has been proposed that decreased levels of DNase activity may be responsible for the persistence of immune complexes in the basement membrane, allowing disease progression [29]. In addition, previous studies have demonstrated that engineered variants of human DNase I can dissociate DNA antibody immune complexes and proposed that this could provide protection against some of the pathological effects of these complexes in the glomeruli [31,32]. Therefore, studies in mice that develop a more severe immune complex mediated glomerulonephritis will be required to test these hypotheses.

In addition to the mouse models of SLE, two studies have been carried out in patients to test the therapeutic effect of recombinant human DNase I [27,28]. Repeated injections were well tolerated but the protein had a short half-life within the periphery and gave no clinical remission. The results presented here suggest that actin/salt-resistant and hyperactive variants of human DNase I, previously shown to have improved activity *in vitro *and in the digestion of mucus from cystic fibrosis patients [31], provide greater protection than the wild-type DNase I and could be considered as a potential therapeutic approach. In addition, the clinical manifestations are more complex in human SLE, with multiple organ involvement, whereas in mice glomerulonephritis is the main disease phenotype. Therefore, extended human trials of human DNase I will provide insights into its potential protective properties in a wider range of tissues.

## Conclusion

This is the first study to directly test the *in vivo *effect of DNase I on the development of lupus-like disease through transgenic protein expression. Our findings indicate that the presence of a mutant DNase (ash.DNase I), resistant to inhibition by serum G-actin, resistant to inhibition by physiological saline and hyperactive compared to the wild-type protein, can provide protection from the development of anti-DNA antibodies, but not renal disease. These results are in line with previous reports on the possible clinical benefits of recombinant DNase I treatment in SLE, and extend them further to the use of engineered DNase I variants with increased activity and resistance to physiological inhibitors.

## Abbreviations

AEU = arbitrary ELISA units; ash.DNase I = actin-resistant, salt-resistant and hyperactive mutant of DNase I; BSA = bovine serum albumin; DNA-MG = DNA-methyl green; dsDNA = double-stranded DNA; ELISA = enzyme-linked immunosorbent assay; G-actin = globular actin; LPS = lipopolysaccharide; PBS = phosphate-buffered saline; SLE = systemic lupus erythematosus; ssDNA = single-stranded DNA; wt.DNase I = wild-type DNase I.

## Competing interests

RAL is an employee of Genentech, Inc., which markets recombinant human DNase I (dornase alfa) under the trade name Pulmozyme^®^.

## Authors' contributions

MB, MJW and PJL conceived the study, acquired the funding, supervised the project's implementation and helped to draft the manuscript. APM performed most of the analysis and compiled the manuscript. FC performed part of the immunoassays. HTK carried out the renal histological analysis. RAL contributed to the development of some of the assays, to the interpretation of the data and helping with the manuscript draft. RF helped to design the transgenic models. All authors have read and approved the final submitted manuscript.

## Figures and Tables

**Table 1:** Analysis of cell populations by flow cytometry

	C57BL/6 (*n *= 3)	ash.DNase I (*n *= 5)	*P*
Spleen			
CD4+ of all lymphocytes (%)	18.1 ± 1.4	18.0 ± 1.6	NS
B/T ratio	6.3 ± 0.2	5.7 ± 1.0	NS
CD4/CD8 ratio	1.6 ± 0.1	1.5 ± 0.2	NS
CD4+ expressing CD62L+ (%)	73.2 ± 2.6	72.8 ± 3.1	NS
Thymus			
CD4/CD8+ lymphocytes (%)	81.6 ± 1.5	79.8 ± 2.4	NS
CD4^+ ^CD8- lymphocytes (%)	3.6 ± 0.3	3.9 ± 0.4	NS

ash.DNase I = actin-resistant, salt-resistant and hyperactive mutant of DNase I; NS, not significant.

**Table 2:** Autoantibody levels in serum of 12-month old mice

Antibody	*Apcs*-/- (*n *= 44)	wt.DNase I. *Apcs*-/- (*n *= 21)	ash.DNase I. *Apcs*-/- (*n *= 21)
Anti-nuclear^a^	1,280 (0–10,240)	2,560 (0–20,480)	320 (0–20,480)
Anti-dsDNA^b^	16.0 ± 8.9	9.2 ± 5.4	5.3 ± 3.5
Anti-chromatin^b^	8.8 ± 2.1	16.1 ± 5.1	10.6 ± 3.9
Anti-histone^b^	56.7 ± 15.2	71.9 ± 17.0	18.6 ± 3.2 (*p *= 0.013)
Anti-ssDNA^b^	12.7 ± 2.7	10.1 ± 3.4	5.2 ± 2.1 (*p *= 0.008)

^a^Values are stated as medians (range in parentheses) based on end-point dilution. ^b^Antibody levels are expressed as arbitrary ELISA units (AEU) relative to a standard positive sample that was assigned a value of 100 AEU. The data are presented as mean ± standard error of the mean. Statistics were performed comparing autoantibody levels from transgenic mice to the control *Apcs*-/- mice, as stated in Materials and methods. Unless stated, there were no significant differences (*p *> 0.05) between the groups. dsDNA, double-stranded DNA; ssDNA, single-stranded DNA.

**Table 3:** Renal histological assessment in 12-month old mice

	Histology grade^a^					
	0	I	II	III	IV	Total

*Apcs*-/-	8 (18)	18 (41)	12 (27)	5 (11)	1 (2)	44
wt.DNase I. *Apcs*-/-	4 (19)	8 (38)	5 (24)	3 (14)	1 (5)	21
ash.DNase I. *Apcs*-/-	4 (19)	7 (33)	5 (24)	5 (24)	0 (0)	21

^a^Values represent the number of mice (%) scored within that grade of glomerular hypercellularity. Grades 0 to IV are as described in Materials and methods. ash.DNase I, actin-resistant, salt-resistant and hyperactive mutant of DNase I; wt.DNase I, wild-type DNase I.
